# Predictive role of PD-L1 expression in the response of renal Medullary carcinoma to PD-1 inhibition

**DOI:** 10.1186/s40425-017-0267-9

**Published:** 2017-08-15

**Authors:** Quaovi Sodji, Kandy Klein, Kavuri Sravan, Jigarkumar Parikh

**Affiliations:** 1 Department of Medicine, Augusta University, 1120 15th St, Augusta, GA 30912 USA; 2 Department of Radiology, Augusta University, 1120 15th St, Augusta, GA 30912 USA; 3 Department of Pathology, Augusta University, 1120 15th St, Augusta, GA 30912 USA; 4 Hematology oncology section, Augusta University, 1120 15th St, Augusta, GA 30912 USA

**Keywords:** Renal medullary carcinoma, Sickle cell trait, Immunotherapy, Pd-L1, Nivolumab

## Abstract

**Background:**

Renal medullary carcinoma is one of the rarest malignancies arising from the kidney. Despite various aggressive therapeutic regimens, mortality remains significantly high (95%) with a median overall survival of 5 months. Furthermore, the scarcity of this malignancy renders randomized clinical trials impossible. We examined the expression of programmed death ligand 1 (PD-L1) in two new renal medullary carcinoma cases, investigated their responses to the PD-L1 inhibitor nivolumab and explored the predictive role of the rate of PD-L1 expression in such response.

**Case presentation:**

Two African-American patients (male and female) with sickle cell trait who presented to our center with hematuria and flank pain were diagnosed with metastatic renal medullary carcinoma. PD-L1 was expressed at rate of 25% and 60% in patient 1 and 2 respectively. Following nephrectomy, they were started on nivolumab. Patient 1 initially responded to the treatment with regression of metastatic lesions. However, following this early response, patient 1 who has been receiving nivolumab for more than 15 months, was noted to have a disease progression. Patient 2 had disease progression after 3 months of nivolumab therapy.

**Conclusions:**

Although PD-L1 is expressed in these patients with renal medullary carcinoma, response to nivolumab was only observed in patient 1 whose tumor has the lowest rate of PD-L1 expression. This may suggest that in RMC, response to PD-L1 inhibition therapy may not correlate with the rate of PD-L1 expression.

## Background

First described in 1995 by Davis et al., renal medullary carcinoma (RMC) is a rare and aggressive malignancy almost exclusively observed in individuals with sickle cell trait (SCT) and sickle cell disease (SCD) [1]. Due to its scarcity, RMC remains the topic of only case reports and small series reports as published by Iacovelli and Alvarez [2, 3]. It mostly arises in the right kidney and as suggested by its nomenclature, from the renal medulla where red blood cells’ sickling is prominent [3]. To date, RMC has been reported in predominantly patients of African descent although there have been cases described in Caucasians and Han Chinese patients [3–5]. Such predominance in the African descent population is due to the prevalence of SCT and SCD, 1/12 and 1/500 respectively [6]. Furthermore, a literature review published by Alvarez et al., revealed a male predominance (70%) with RMC and 88.6% of patients had the sickle cell trait (AS genotype) whereas only 2.3% had SCD (SS genotype) [3]. The most common symptoms at presentation include hematuria and pain (67%), weight loss (23%) and respiratory distress secondary to mass effect or pleural involvement [2]. Due to the aggressive nature of this malignancy, most patients present with metastatic lesions primarily to lymph nodes, lungs, liver, adrenal glands and bone. Moreover, the primary tumor size at presentation is greater than 4 cm [3]. Despite current treatments which include nephrectomy and various chemotherapy regimens, the overall mortality of RMC remains significantly elevated due to metastatic disease at presentation, resistance to chemotherapy and radiation therapy (RT) [4]. The overall survival (OS) is estimated at 17.0 months and 4.0 months in localized and metastatic disease respectively with median OS of 5.0 months, highlighting the need for novel therapies [2].

Recent advances in cancer immunology have demonstrated the crucial role of the immune system in cancer progression, resulting in the identification of multiple therapeutic targets and the development of novel immunotherapy drugs [7]. Nivolumab, a PD-L1 inhibitor, has been effective against various malignancies in pre-clinical studies and clinical trials with a relatively favorable toxicity profile. Such efficacy was demonstrated in the CheckMate 025 trial where patients with advanced renal cell carcinoma with clear cell histology on nivolumab had a 25.0 months median overall survival compared to 19.6 months in patients on everolimus. Furthermore, nivolumab had fewer grade 3 or 4 side effects compared to everolimus [8]. Currently, nivolumab is FDA approved for the treatment of non-small cell lung cancer, metastatic melanoma, squamous cell carcinoma of the head and neck, renal cell carcinoma, classical Hodgkin lymphoma and urothelial carcinoma with ongoing clinical trials to broaden its therapeutic use against other malignancies. Recently, Beckermann et al. reported the use of nivolumab in a patient with RMC [9].

Herein, we report the expression of PD-L1 in two RMC patients, their responses to PD-L1 inhibition with nivolumab and the predictive role of the level of PD-L1 expression in such response.

## Case presentation

### Patient 1

**Fig. 1:**
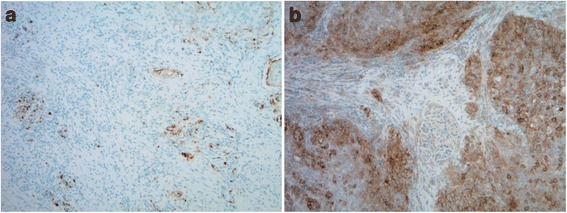
PD-L1 expression in patients with Renal medullary carcinoma (RMC). **a** Patient 1, PD-L1 SP142 (25% Immune cells stained (ICS); reference/Positive: ICS ≥ 5%). **b** Patient 2, PD-L1 IHC 28–8 (60% tumor cells stained (TCS); reference/Positive: TCS ≥ 1%)

**Fig. 2:**
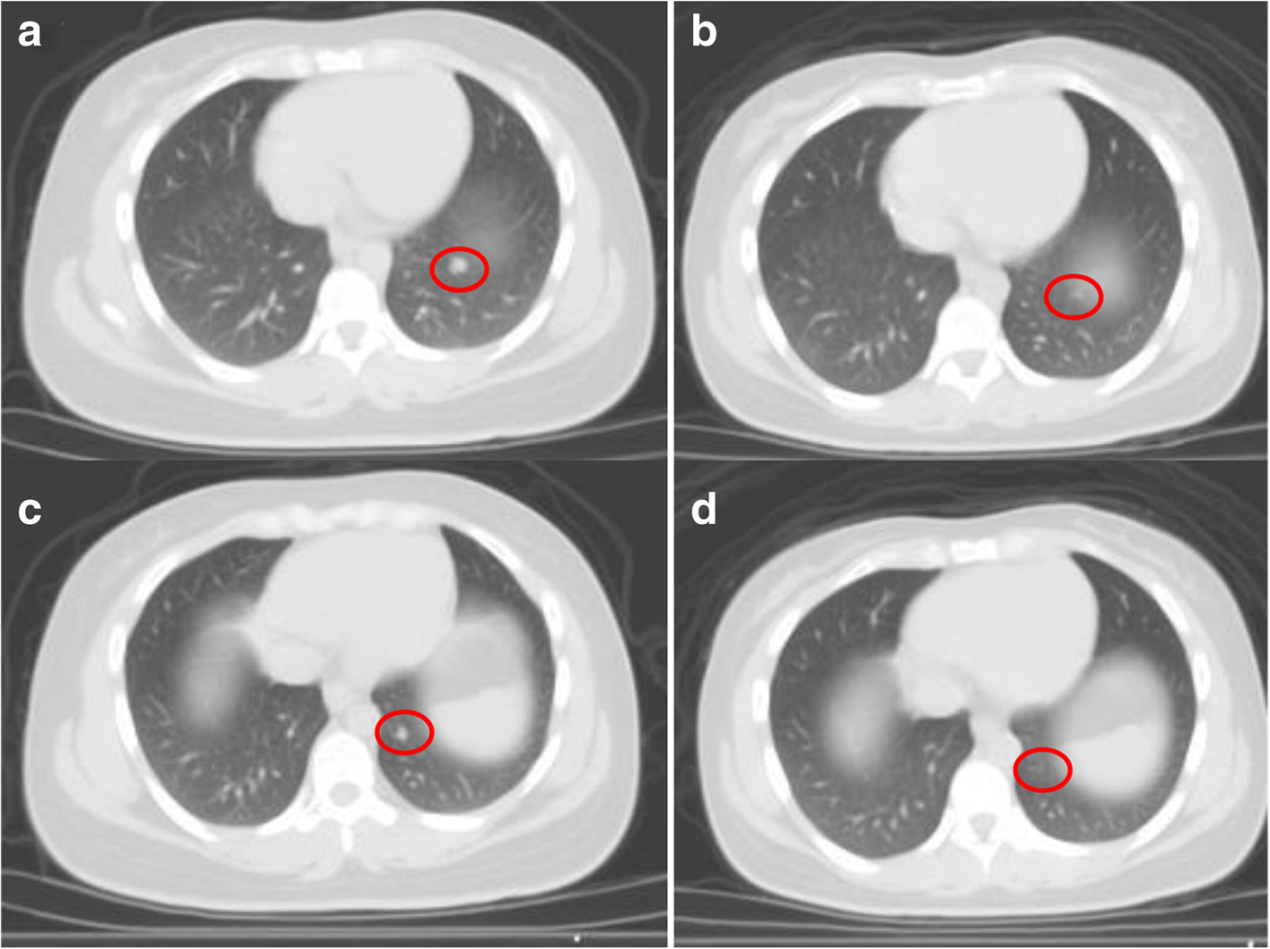
Axial CT demonstrating interval improvement in the metastatic lung lesions in patient 1 after 5 cycles of Nivolumab. **a** Left lower lobe nodule #1 prior to Nivolumab. **b** Left lower lobe nodule #1 after Nivolumab. **c** Left lower lobe nodule #2 prior to Nivolumab. **d** Left lower lobe nodule #2 after Nivolumab

**Fig. 3:**
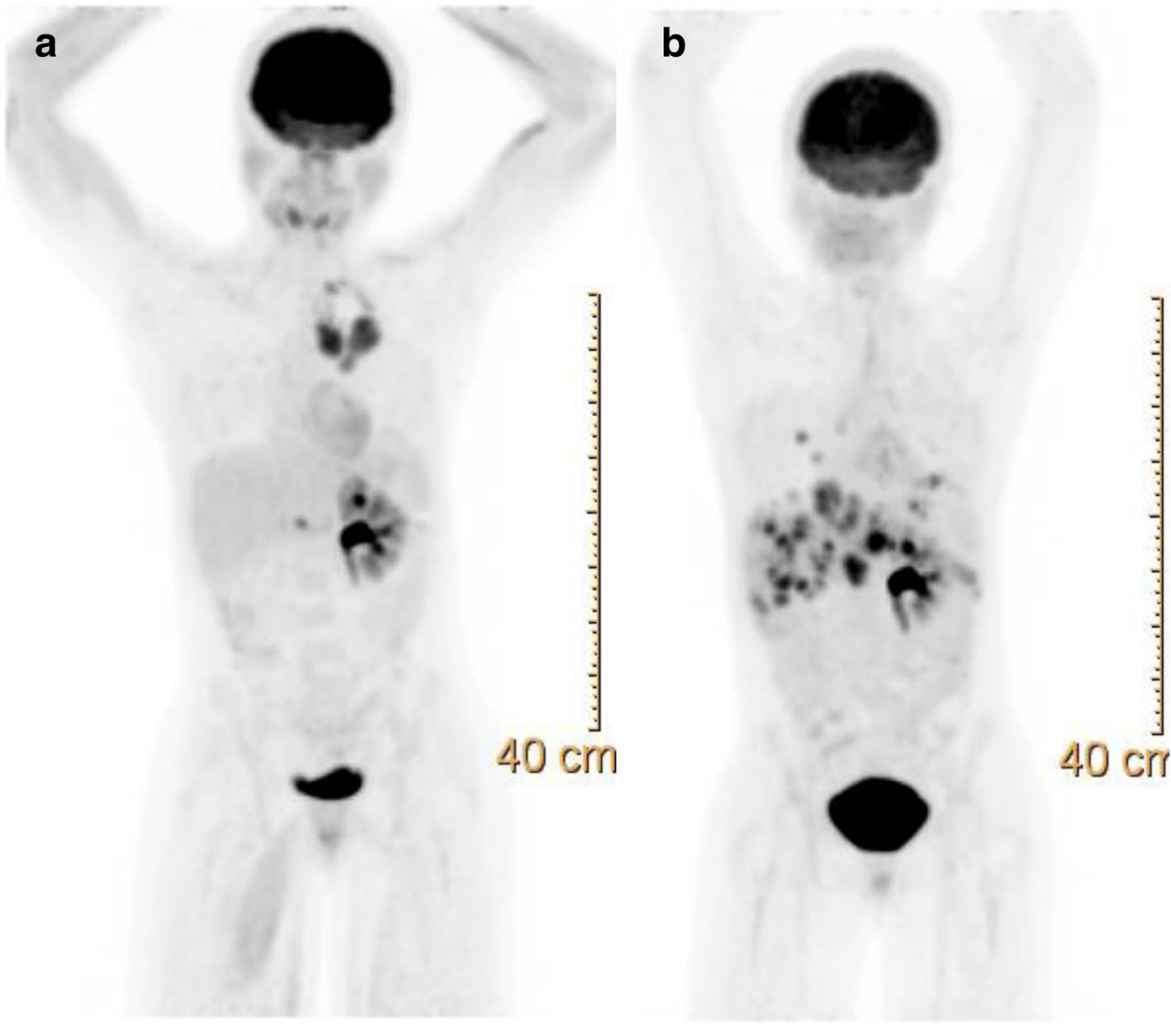
PET scan of patient 1 demonstrating disease progression. **a** Scan obtained in October 2016 showing prominent mediastinal lesions. **b** Latest scan in February 2017 after 28 cycles of nivolumab and palliative radiation to the mediastinum showing new metastatic lesions in the liver and lungs but resolution of medistanial disease

The patient is a 24 year-old African-American female with SCT who presented with a one month history of gross hematuria and intermittent right flank pain in December 2014. Computed tomography (CT) imaging revealed a well-defined solid mass with a central necrosis within the upper pole of the right kidney measuring 6.0 cm × 3.9 cm × 5.0 cm and two pulmonary nodules (3 and 2 mm in size) in the left lower lobe concerning for metastatic disease. Thus in January 2015, right radical nephrectomy was performed. Histological and Immunohistochemical (IHC) analysis revealed RMC. Further genetic study revealed SMARCB1 mutation as previously reported in RMC [10]. Follow up imaging in February 2015 showed an interval increase in the size of the aforementioned pulmonary nodules to 7 mm and a new lesion in the upper left lobe. Unfortunately, two new nodules of 1 cm each were also detected in the post-surgical bed. Subsequently in March 2015, she was started on palliative chemotherapy consisting of cisplatin, paclitaxel and gemcitabine. Due to a grade 3 neutropenia she experienced after the first cycle, she only received cisplatin and gemcitabine for the remainder of the treatment. After the third cycle of the cisplatin and gemcitabine, a complete response was observed and at the end of the sixth cycle in July 2015, she remained free of disease. A follow up CT abdomen and pelvis performed in October 2015 showed an increased in disease burden with two pulmonary nodules in the left lower lobe and a 1.6 × 1.6 cm nodule within the right nephrectomy bed. IHC staining performed on the initial nephrectomy specimen revealed the expression of PD-L1 on 25% of tumor cells (Fig. 1a). As such, she was started on nivolumab 3 mg/kg every 2 weeks on October 19th 2015. She tolerated well the infusion of nivolumab except for mild nausea controlled with ondansetron. After completing 4 cycles of nivolumab, she presented at an outside hospital in December 2015 for UTI and was found to be 6 weeks pregnant prompting the nivolumab to be stopped. She elected to undergo a dilation and curettage in January 2016. From November 30th 2015 (date of her 4th cycle), to February 12th 2016 (5th cycle of nivolumab), she did not receive nivolumab. Prior to resuming the nivolumab, a CT scan showed a stable disease in the right nephrectomy bed and improved metastatic lung disease (Fig. 2). She received 8 additional cycles of nivolumab before undergoing another PET scan in May 2016 which showed a slight increase in the recurrent lesion in the surgical bed and a persistent resolution in metastatic lesions to the left lower lung lobe. Despite the interval progression in the surgical bed, she remained asymptomatic. In July 2016 she presented to the emergency room with nausea, diarrhea and abdominal pain suggestive of colitis. An improvement was noted when with a course of prednisone. While she was recovering from the colitis, the nivolumab was stopped. She underwent a surgical resection of the recurrent lesion and resumed the nivolumab on July 21st 2016 (Cycle#13). After receiving 5 additional cycles, a PET scan in October 2016 revealed an increase in the disease burden. She received palliative radiation to the left mediastinal disease with 4400 cGy in 200 cGy fraction and to the left upper lung lobe lesion with 6000 cGy in 200 cGy fraction via intensity-modulated radiation therapy (IMRT) while continuing nivolumab. She completed the radiation therapy in December 2016 and continued to receive nivolumab. Since the initiation of nivolumab, the patient has completed 28 cycles and a follow up PET scan in February 2017 showed a decreased in tumor burden in the mediastinum but further progression outside of the radiation field with new lung and liver lesions and recurrence in the right nephrectomy bed (Fig. 3). In light of disease progression, the decision was made to add ipilimumab to the nivolumab. At time of this submission, response assessment from this therapy is awaited (See Table 1 for a summary of chemotherapy and immunotherapy agents administered).

**Table 1:** Summary chemotherapy and immunotherapy agents received by patient 1

Treatments	Duration
Cisplatin, paclitaxel^a^, gemcitabine	Six cycles
Nivolumab	28 cycles (56 weeks)^b^
Ipilimumab	2 cycles (6 weeks)^c^

^a^Paclitaxel was stopped after first cycle due to grade 3 toxicity

^b^At the time of submission of this manuscript, 28 cycles were completed but nivolumab will be continued

^c^At the time of submission of this manuscript, 2 cycles were given and will be continued for 2 more cycles

### Patient 2

The patient is 57 year-old African-American male with SCT who presented with hematuria and right flank pain. Subsequent CT scan revealed a mass on the upper pole of the right kidney. He underwent in July 2016 a right nephrectomy which revealed a tumor size of 7.4 cm × 4.8 cm × 4.8 cm and RMC histology. As seen in patient 1, PD-L1 was expressed on 60% of this patient’s tumor (Fig. 1b). A PET scan in August 2016 revealed an extensive retroperitoneal lymphadenopathy. On September 9th 2016, he was started on nivolumab 3 mg/kg every 3 weeks. He tolerated nivolumab infusion well and after the fifth cycle, a PET scan was performed. Unfortunately, disease progression was noted in the nephrectomy bed along with new metastatic lesions to the lungs, mediastinum and retroperitoneum.

## Discussion

Despite very aggressive treatments, RMC still has a very high mortality rate. Treatments include nephrectomy and chemotherapy [11]. Patients who had nephrectomy had a 6.0 months survival compared to 3.0 months in patients who did not [2]. The very low prevalence of RMC has made randomized trials impossible. As such, most chemotherapy regimen used today are the results of retrospective studies or anecdotal findings. The first line of chemotherapy agents include platinum-based chemotherapy such as cisplatin or carboplatin, paclitaxel and gemcitabine regimen (CPG) or methotrexate, vinblastine, Adriamycin (Doxorubicin) and cisplatin regimen (MVAC) [12, 13]. Other chemotherapy drugs such bortezomib, topotecan and bevacizumab have also been reported [14, 15]. Although patients treated with the CPG regimen had longer OS (12.0 months) compared to those receiving the MVAC regimen (4.0 months), the difference was not statistically significant. Based on the aforementioned literature findings, patient 1 was started on the CPG regimen following the right nephrectomy. However, due to the grade 3 neutropenia she only received cisplatin and gemcitabine to which she had a complete response after 6 cycles. Aware of the high recurrence rate of the RMC, we decided to explore other therapeutic avenues. Recently, multiple trials have been exploring the use of immunotherapy agents such as nivolumab in clear cell renal carcinoma [16]. Such trials were guided by IHC studies showing the expression of PD-L1 [17]. Furthermore, in a study conducted by Choueiri et al. on nonclear-cell renal cell carcinoma (RCC), PD-L1 expression was found in various renal carcinomas (chromophobe RCC, papillary RCC, Xp11.2 translocation RCC and collecting duct carcinoma) with the collecting duct and X11p translocation having an expression rate of 20 and 30% respectively [18]. The aforementioned study did not include RMC. Subsequently, we examined the PD-L1 expression in both patients’ tumor. IHC staining (Fig. 1) revealed the expression of PD-L1, 25% of tumor cells in patient 1 versus 60% in patient 2, suggesting a potential therapeutic benefit for PD-L1 inhibition. Initially, the first patient responded to the treatment with regression in the metastatic lesions and a stable disease in the nephrectomy bed (Fig. 2). However, she had multiple treatment interruptions resulting in the nivolumab not being administered as scheduled for extended period. Surprisingly, patient 2 did not respond to nivolumab despite a higher expression rate of PD-L1 than patient 1. He had significant disease progression after completing 5 cycles of nivolumab.

Beckermann et al. recently reported a complete response to nivolumab in a patient with RMC whose tumor also expressed PD-L1 [9]. In such patient, PD-L1 was expressed in 23% of tumor cells compared to 25 and 60% in the patients described in this report. Altogether, these findings suggest that in RMC, increasing expression of PD-L1 may be associated with poor prognosis as observed in clear cell and nonclear-cell RCC [8, 18]. However, such expression level does not appear to correlate with clinical response to PD-L1 inhibition. This observation suggests that in RMC the level of PD-L1 expression is not predictive of the clinical response to PD-L1 inhibition.

## Conclusion

We reported the expression of PD-L1 in two patients with RMC and their responses to nivolumab. The choice of this therapy was based on the expression of the PD-L1 and on the relatively safe toxicity profile of nivolumab. Although in these patients PD-L1 was expressed, response to nivolumab was only observed in the patient with the lower expression level of PD-L1. This suggests that in RMC, PD-L1 expression may not be the sole predictive marker for response to PD-L1 inhibition therapy. As suggested by Hugo et al. in melanoma, additional genomic and non-genomic makers may be involved in RMC response to PD-L1 inhibition [19]. Furthermore, the aggressive nature of this malignancy and metastasis at the time of presentation may also hinder the response to PD-L1 inhibition. It is also possible that RMC patients presenting with localized disease or with minimal metastatic disease may have an optimal benefit from nivolumab treatment. PD-L1 expression has been evaluated in only a few patients with RMC due to the scarcity of this renal malignancy. Evaluating the expression of PD-L1 in newer cases of RMC will be of great value in understanding its prognostic role and predictive value in the response to PD-L1 inhibition therapy.

## Abbreviations

IHC: Immunohistochemical
IMRT: Intensity-modulated radiation therapy
OS: Overall survival
PD-L1: Programmed death-ligand 1
RCC: Renal cell carcinoma
RMC: Renal medullary carcinoma
RT: Radiation therapy
SCD: Sickle cell disease
SCT: Sickle cell trait
